# Eczematous Neurodermatitis Following Total Knee Arthroplasty: Expanding Awareness of a Rare and Underdiagnosed Postoperative Dermatologic Complication

**DOI:** 10.7759/cureus.86989

**Published:** 2025-06-29

**Authors:** Eliana Jolkovsky, Erik Zeegen, Iris Ahronowitz

**Affiliations:** 1 Dermatology, University of California Los Angeles David Geffen School of Medicine, Los Angeles, USA; 2 Orthopedic Surgery, University of California Los Angeles David Geffen School of Medicine, Los Angeles, USA

**Keywords:** autonomic denervation dermatitis, eczematous neurodermatitis, postoperative dermatitis, skinted, total knee arthroplasty

## Abstract

SKINTED (surgery of the knee, injury to the infrapatellar branch of the saphenous nerve, traumatic eczematous dermatitis) is a recently described dermatological condition resulting from injury to the infrapatellar branch of the saphenous nerve (IPBSN) during knee surgery. This rare and underdiagnosed surgical complication characteristically presents as an eczematous eruption within an area of altered skin sensation in the distribution of IPBSN innervation. We describe a case of SKINTED in a 64-year-old female patient who presented with localized dermatitis, severe pruritus, intermittent pain, and mild numbness of her knee six months following total knee arthroplasty. Dermatologic evaluation led to a clinical diagnosis of SKINTED, and treatment with topical corticosteroids successfully resolved her symptoms. This case reinforces the need to consider SKINTED in appropriate patients. Increased awareness of this condition among both dermatologists and orthopedic surgeons can prevent misdiagnoses and unnecessary use of antibiotics, biopsies, and imaging, as well as help alleviate patient anxiety.

## Introduction

The first case report of neurodermatitis following total knee arthroplasty (TKA) was published in 1993 by Satku et al. [1]. The name SKINTED (surgery of the knee, injury to the infrapatellar branch of the saphenous nerve, traumatic eczematous dermatitis) was later coined in 2009 by Verma and Mody [2] and remains a rarely diagnosed dermatologic condition. SKINTED is an autonomic denervation dermatitis (ADD) affecting the skin innervated by the infrapatellar branch of the saphenous nerve (IPBSN), a purely sensory nerve. This neurocutaneous complication of knee surgery typically presents as eczematous dermatitis (ill-defined erythematous and scaly patch of skin), often in an area of hypoesthesia or anesthesia on the knee. The exact pathogenesis is unknown. It is hypothesized that localized denervation due to iatrogenic IPBSN injury leads to cutaneous immune dysregulation, similar to proposed mechanisms for post-herpetic dermatitis [3]. Importantly, SKINTED can present with only subtle sensory changes, including mild numbness or intermittent pain. SKINTED most commonly presents within three to six months after surgery, with an incidence of approximately 5% [4]. As a rare complication of TKA, SKINTED can be misdiagnosed as allergic contact dermatitis (including raising concerns of hypersensitivity to prosthetic implant materials), fungal infection, or cellulitis. Recognition of SKINTED, which responds well to topical steroid therapy [4], can prevent unnecessary use of antibiotics, imaging, biopsy, or unnecessary surgical revision. We present a case of SKINTED with severe pruritus, mild numbness, and intermittent pain in a 64-year-old female patient six months following TKA of her right knee. Our report highlights the importance of recognizing SKINTED even when sensory changes are subtle, emphasizing the need for clinical awareness of this rare postoperative complication.

## Case presentation

A 64-year-old female patient (body mass index: 32 kg/m^2^) with a medical history of functional diarrhea, gastric metaplasia, Gilbert’s disease, myocardial bridge, coronary artery disease, and cervical radiculopathy, underwent medial parapatellar approach right TKA for severe osteoarthritis. Her only prior orthopedic surgery was an uncomplicated and successful left knee arthroscopic debridement with a lateral patellar retinacular release, performed 13 years earlier for a microfracture of the medial femoral condyle. One month before the onset of SKINTED, the patient underwent a sacral nerve stimulator trial for treatment-refractory fecal incontinence. The incontinence was attributed to an anal sphincter injury during polyp removal via colonoscopy several years prior.

Her allergy history included a moderate contact allergy to adhesive bandage (erythema) and skin blistering from allergy to mastisol adhesive strips, which were not used after this procedure. The patient's wound closure was with skin staples without any skin adhesive and then covered with a silicone silver impregnated dressing. Importantly, she had no history of metal sensitivity or other relevant allergies that might suggest an allergic reaction to prosthetic components. Her family history was notable for basal cell carcinoma and squamous cell carcinoma in her father and Crohn’s disease in her mother and brother. The patient had never smoked or used smokeless tobacco or other recreational drugs. She reported consuming one to two standard drinks of alcohol per week.

Her postoperative course was largely unremarkable aside from transient knee pain on postoperative day 10, which was managed with over-the-counter analgesics. By seven weeks after surgery, the patient had completed physical therapy and was ambulating independently without an assistive device, indicating good functional recovery. She denied fevers, wound drainage, or other signs of infection. On examination, the surgical scar had healed without signs of infection. At the time, she was taking methocarbamol 500 mg twice daily for persistent muscle spasms but did not require any other pain medications. Six months postoperatively, a right knee X-ray showed an intact TKA with anatomic alignment and no evidence of complications. A small, nonspecific joint effusion was noted (felt not to be clinically significant), and no acute osseous abnormalities were identified.

Five and a half months following TKA, the patient developed a dry, thickened, intensely pruritic, and intermittently painful patch of skin. The affected area was on the inferolateral aspect of the surgical knee, below the patella and lateral to the incision site. She reported accompanying numbness in the affected skin. Over-the-counter hydrocortisone cream did not improve her symptoms, and the patient was referred by her orthopaedic surgeon for a dermatological evaluation.

**Figure 1:**
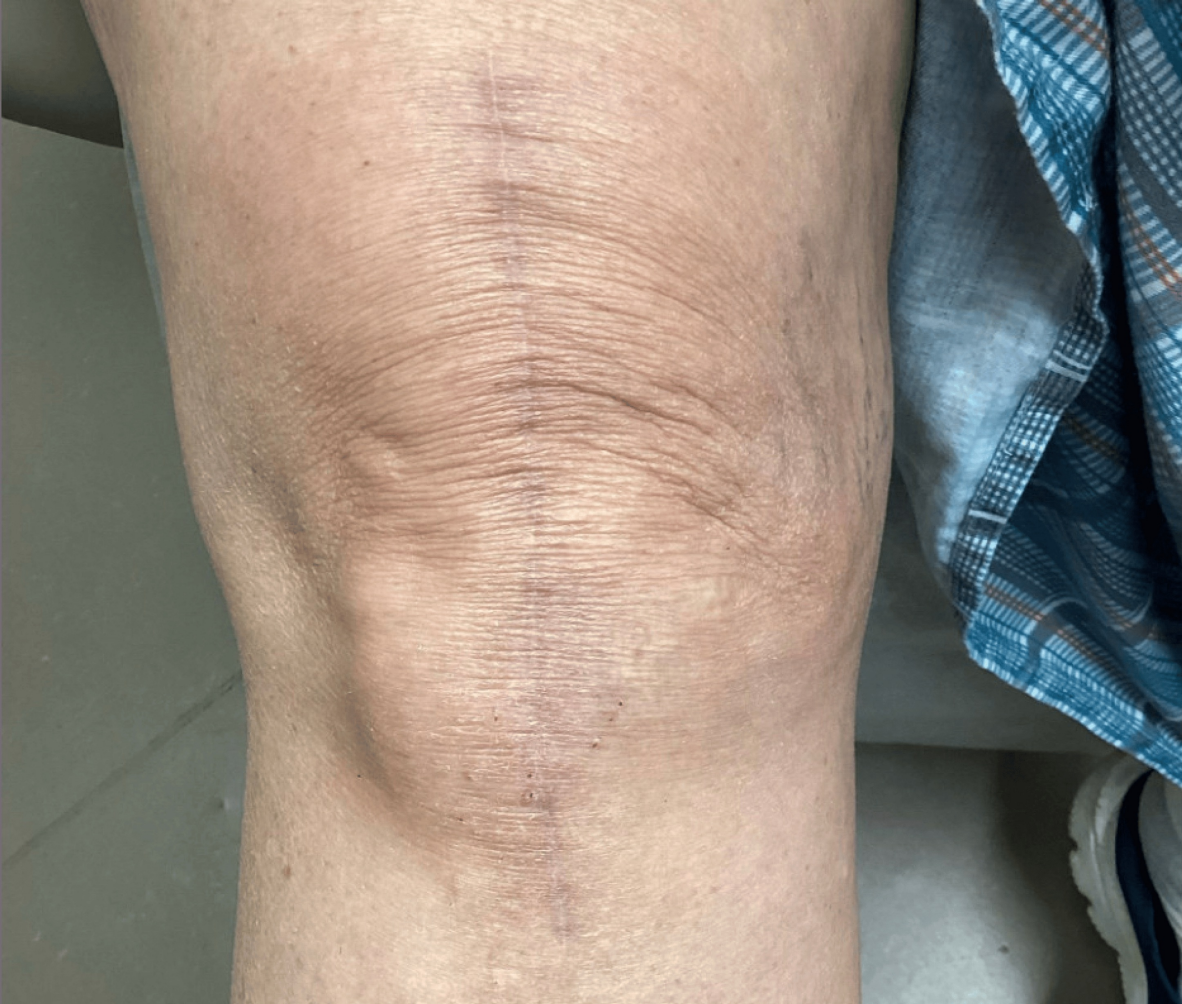
Photograph taken at the dermatology clinic of a well-healed scar six months after total knee arthroplasty, with an ill-defined, mildly scaly, erythematous patch at the inferolateral border.

**Figure 2:**
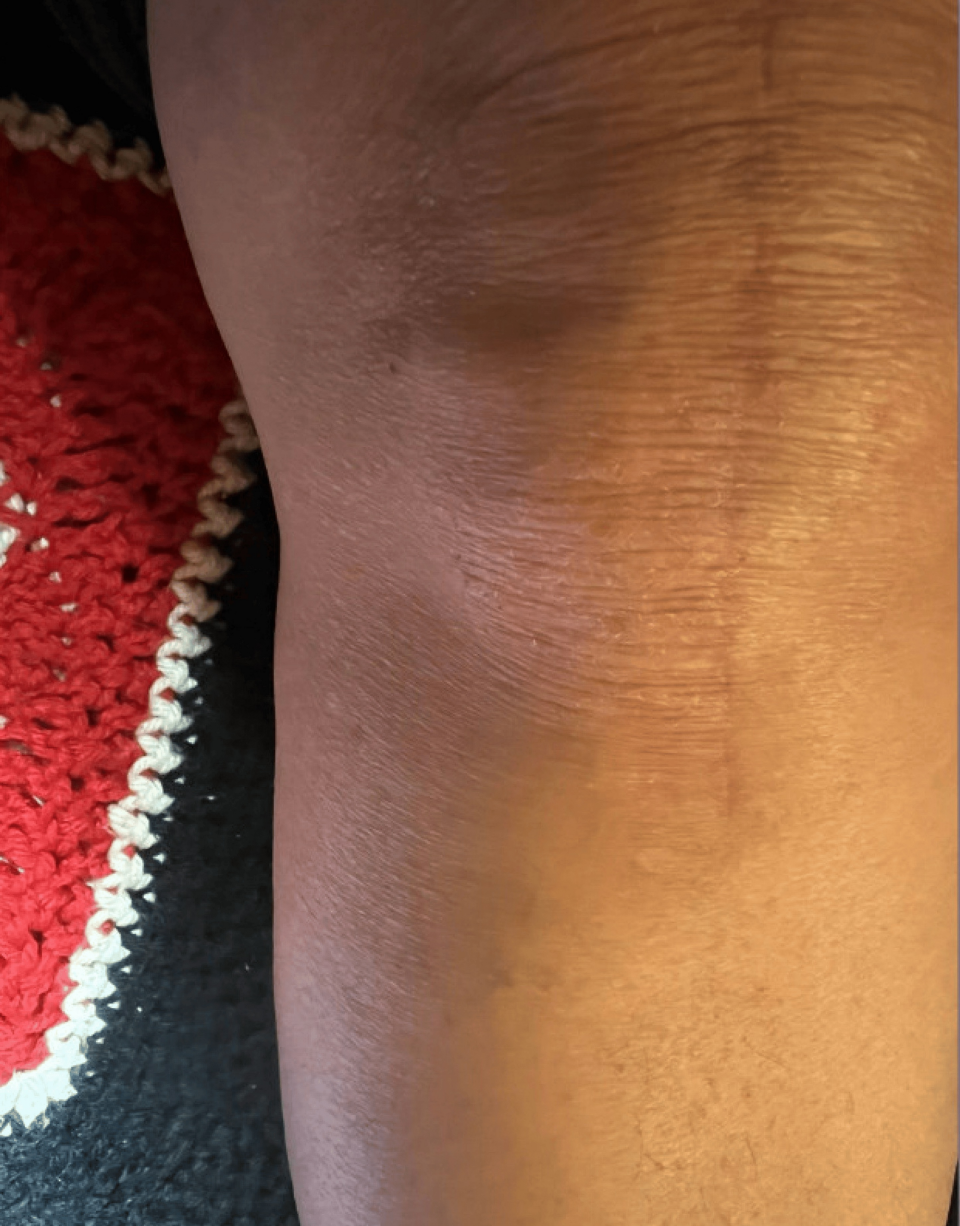
Patient-submitted photograph demonstrating the same localized eczematous patch at the inferolateral border of the incision scar.

Upon dermatologic evaluation two weeks later, an ill-defined, focal, erythematous, eczematous patch measuring approximately 4 cm in width and 5 cm in length was present at the inferolateral edge of a well-healed surgical scar. The patient described an “insatiable itch” in the same quadrant of her knee, accompanied by numbness. She reported that the pain was tolerable, but the itching was significantly more intense and distressing. It was often triggered by incidental contact, such as brushing against bed covers. Photographs obtained at this visit and submitted by the patient demonstrated the inferolateral patch with subtle erythema (Figures 1, 2). The patient was otherwise asymptomatic, aside from pre-existing functional diarrhea. At that time, her outpatient medications included metoprolol succinate, oral progesterone, transdermal estradiol, oral glucosamine-chondroitin, vitamin D, ascorbic acid, and docusate. As-needed medications included ondansetron and tramadol, neither of which was being taken regularly. None of these medications was suspected to be implicated in her cutaneous findings. She was not on systemic corticosteroids or immunosuppressive therapy. A clinical diagnosis of SKINTED was made, and the patient was started on a trial of 0.1% triamcinolone ointment applied to affected areas twice daily. No labs, biopsies, cultures, or imaging studies were ordered.

Within three weeks after starting topical corticosteroid therapy, the patient reported significant improvement in her rash, itching, and numbness with no new areas of involvement, and no longer required triamcinolone. However, on occasion, she experienced self-limiting episodes of pruritus in the affected area even in the absence of visible skin changes. At the three-month follow-up, the patient reported persistent minor numbness and occasional brief episodes of deep itching in the affected area, none of which required further treatment.

## Discussion

According to the American Academy of Orthopaedic Surgeons, approximately 2.1 million TKA surgeries were performed between 2012 and 2023 in the United States [5]. SKINTED is a rare dermatological complication of knee surgery. It results from iatrogenic injury to the IPBSN and typically presents with eczematous dermatitis localized to the knee in an area of hypoesthesia or anesthesia [1,2]. The IPBSN is a purely sensory nerve that provides cutaneous sensation to the anterior knee and innervates the anterior-inferior joint capsule. Due to its superficial course across the knee, the IPBSN is vulnerable to injury during TKA [6]. SKINTED has also been reported following other types of knee surgery, such as arthroscopy or saphenous vein grafting [7,8].

Clinically, SKINTED usually presents as a well-demarcated, scaly, erythematous, or hyperpigmented patch or plaque in an area of altered sensation on the anterior knee, most often lateral to the surgical incision. Nazeer et al. (2020) reported an incidence of 4.4% (9 cases out of 203 TKA procedures) with a mean time to diagnosis of four months postoperatively [4]. Similarly, Mukartihal et al. (2023) reported an incidence of 5.52% in a cohort of 3,318 TKA patients, with a mean duration to diagnosis of 4.4 months postoperatively [9]. The size of the affected area of skin ranged from 3 cm × 2 cm to 12 cm × 10 cm at the time of initial dermatologic evaluation [4]. The mean time to resolution of dermatitis with topical steroid therapy was 7.7 weeks. In most cases, diagnoses were made clinically (based on a delayed onset of morphologically and topographically typical dermatitis within a zone of altered skin sensation), without histopathological examination or laboratory studies. None of the patients experienced any other dermatological or surgical postoperative complications before the onset of neuropathic dermatitis. A summary of findings in previously reported case series is included (Table 1).

**Table 1:** Summary of prior case series reporting surgery of the knee, injury to the infrapatellar branch of the saphenous nerve, and traumatic eczematous dermatitis. TKA: total knee arthroplasty

Reference	Number of cases/% incidence	Demographics	Presentation (timing, location, ancillary findings)	Treatment	Follow-up (treatment response, recurrence)
Verma and Mody (2009) [2]	55 cases (case series, incidence not given)	16 cases, including 11 men (68.75%), 5 women (31.5%), available for regular follow-up	Onset three weeks to four months after the surgery. Location of the lesions was exclusively lateral in 12 (75%) patients, whereas the other four (25%) patients had involvement of the skin on both sides of the incision. Most patients reported anesthesia/hypoesthesia in the area	Topical emollients and topical steroids	Gradual resolution of all lesions. Some patients noted recurrence or worsening of existing lesions in the winter
Nazeer et al. (2020) [4]	Nine lesions in eight patients were identified out of a total of 203 consecutive TKAs, with an estimated incidence of 4.4%. (single institution retrospective study)	Eight patients (seven women and one man). Mean age: 64.5 years (range: 58–78 years)	Mean time to onset: four months (range: 3–6 months). The eruption exclusively occurred lateral to the midline skin incision. Pruritus over the lesion was reported by five patients. Perilesional and marginal hypoesthesia in all patients. The size of lesions ranged from 3 cm × 2 cm to 12 cm × 10 cm	Midpotency topical steroid cream application twice daily, along with topical emollient application three times daily. Patients with pruritus were prescribed oral antihistamines for five days	Complete response, without recurrence, in all patients at the end of six months. Five of the nine lesions healed completely within six weeks. The remainder healed completely within 10 weeks. All patients were followed for at least six months from the initial presentation. No recurrences
Mukartihal et al. [9]	A total of 3,318 patients with 4,282 TKAs were included, of which 188 patients presented with the clinical picture of neuropathic dermatitis. (183 included in final analysis with 5 lost to follow-up). Calculated incidence of 5.52%. A single-institution retrospective study	136 females and 52 males with a mean age of 67.13 years (range: 37–92 years). Mean body mass index was 28.2 kg.m^2^ (range: 19.4–36.3 kg/m^2^)	Mean onset: 4.4 months (range: 2–6 months)	Mildly potent steroid ointment twice a day and an emollient were prescribed to all patients. Patients with pruritus were also given oral antihistamines for seven days	Mean time to resolution of skin lesions 7.67 (range: 6–12) weeks. In seven patients, healing took a slightly longer period, up to 12 weeks. The minimum follow-up for all patients post-TKA was two years. No recurrence noted. Sensory loss gradually improved in all patients over the period to near normal in 178 patients, though five (2.7%) patients still have a significant level of hypoesthesia

Our patient reported symptoms including severe pruritus, occasional pain, and mild numbness in the area of the eruption. In the study by Nazeer et al., five of the eight (62.5%) patients with SKINTED presented with pruritus. They found that all of the eczematous patches (nine patches total in eight patients) were within an area of hypoesthesia. Likewise, all patients in the study by Mukartihal et al. had altered skin sensation, ranging from mild hypoesthesia to complete sensory loss. As with our patient, full body skin examinations in the Nazeer et al. (n = 8) and Mukartihal et al. (n = 183) studies were unremarkable without any other rashes or lesions of concern. All patients in both studies had well-functioning knees postoperatively. Although other reports of SKINTED describe the condition as consistently associated with hypoesthesia, our case demonstrates that even relatively subtle sensory changes may accompany the condition [1,4,8-12].

In addition to SKINTED, other neurocutaneous conditions, such as post-herpetic dermatitis, illustrate that nerve injury can result in localized immune dysregulation and eczematous skin changes [3]. Although rare and benign, SKINTED warrants further attention as it can go undiagnosed or misdiagnosed by both dermatologists and orthopedic surgeons. In particular, metal hypersensitivity dermatitis, which is typically a diagnosis of exclusion, should be considered and ruled out in the differential diagnosis. Patients with this rare postoperative condition often have knee effusions and synovitis and, less frequently, dermatitis. The rash is erythematous, papular, pruritic, and scaly. Unlike SKINTED cutaneous lesions that appear only lateral to the surgical scar in areas of hypoesthesia, a metal hypersensitivity rash can occur on either side of the scar and may even appear on the trunk, neck, and other extremities.

Our case illustrates that even when sensory symptoms are mild, nerve injury may still cause immune dysregulation and dermatitis. Recognizing and treating SKINTED can help prevent secondary skin infection, avoid unnecessary procedures, and alleviate patient anxiety.

## Conclusions

SKINTED is a rare, benign postoperative dermatologic complication that presents as well-demarcated dermatitis within a zone of altered skin sensation on the anterior knee. Our report contributes to the growing literature on SKINTED by reinforcing that even subtle sensory changes may be associated with this condition. Misdiagnosis can result in unnecessary skin biopsies, imaging studies, or inappropriate therapies. Prompt clinical recognition of SKINTED is helpful, as the condition often requires no further diagnostic workup, and typically improves within weeks to months with topical steroid therapy.
